# Failure of Passive Immune Transfer in Neonatal Beef Calves: A Scoping Review

**DOI:** 10.3390/ani15142072

**Published:** 2025-07-14

**Authors:** Essam Abdelfattah, Erik Fausak, Gabriele Maier

**Affiliations:** 1 Department of Population Health and Reproduction, School of Veterinary Medicine, University of California, Davis, CA 95616, USA; 2 Department of Animal Hygiene and Veterinary Management, Faculty of Veterinary Medicine, Benha University, Moshtohor 13518, Qalyubia, Egypt; 3 University Library, University of California, Davis, CA 95616, USA; edfausak@ucdavis.edu

**Keywords:** beef cattle, colostrum management, dystocia, failure of transfer of passive immunity (FTPI), maternal antibodies, neonatal calves, risk factors, scoping review, vaccination

## Abstract

Neonatal calves possess an immature and naïve immune system and are reliant on the intake of maternal colostrum for the passive transfer of immunoglobulins. Maternal antibodies delivered to the calf via colostrum, are crucial to prevent calfhood diseases and death. Failure of transfer of passive immunity (FTPI) is a condition in which calves do not acquire enough maternal antibodies, mostly in the form of IgG, due to inadequate colostrum quality or delayed colostrum feeding. The diagnosis and risk factors for FTPI have been widely studied in dairy cattle; however, in beef calves, the research interest in the topic is relatively recent, and the most adequate diagnostic and preventative methods are still in development, making it difficult to define recommendations for the assessment and prevention of FTPI in cow–calf operations. The objective of this scoping review is to identify the published literature on best practices for colostrum management and transfer of passive immunity (TPI) in neonatal beef calves. The literature was searched using three electronic databases (CAB Direct, Scopus, and PubMed) for publications from 2003 to 2025. The search process was performed during the period from May to July 2023, and was repeated in January 2025. All screening processes were performed using Covidence systematic review software (Veritas Health Innovation, Melbourne, Australia). A total of 800 studies were initially identified through database searches. After removing duplicates, 346 studies were screened based on their titles and abstracts, leading to the exclusion of 260 studies. The remaining 86 studies underwent full-text screening, and 58 studies were considered eligible for data extraction. Hand-searching the references from published review papers on the subject yielded an additional five studies, bringing the total to 63 included articles. The prevalence of FTPI has been estimated to be between 5.8% and 34.5% in beef calves. Factors studied related to colostrum management include quality and quantity of colostrum intake, the timing and method of colostrum feeding, and the microbial content of the colostrum. Studies on risk factors related to the calf include the topics calf sex, twin status, calf vigor, weight, month of birth, cortisol and epinephrine concentrations, and the administration of nonsteroidal anti-inflammatory drugs to calves after difficult calving. The dam-related risk factors studied include dam body condition score and udder conformation, breed, parity, genetics, prepartum vaccinations and nutrition, calving area and difficulty, and the administration of nonsteroidal anti-inflammatory drugs at C-section. Most importantly for beef systems, calves with low vigor and a weak suckling reflex are at high risk for FTPI; therefore, these calves should be given extra attention to ensure an adequate consumption of colostrum. While serum IgG levels of < 8 g/L or < 10 g/L have been suggested as cutoffs for the diagnosis of FTPI, 16 g/L and 24 g/L have emerged as cutoffs for adequate and optimal serum IgG levels in beef calves. Several field-ready diagnostics have been compared in various studies to the reference standards for measuring indicators of TPI in beef calves, where results often differ between models or manufacturers. Therefore, care must be taken when interpreting these results.

## Simple Summary

Neonatal calves rely on maternal colostrum for immunity, which is crucial for preventing disease and death. Failure of transfer of passive immunity (FTPI) occurs when calves do not absorb enough maternal antibodies, often due to poor colostrum quality or delayed feeding. While FTPI is well-studied in dairy cattle, the interest in researching the topic in beef herds is relatively recent. A scoping review aimed at identifying best practices for colostrum management in beef calves found various risk factors affecting passive transfer success, related to both dam and calf. Low calf vigor and weak suckling reflex increase FTPI risk, requiring special attention. Achieving levels of adequate and optimal serum antibody levels in calves may improve herd health. Various field-ready diagnostics are available, but care must be taken when interpreting the results, because studies show variability between diagnostic modalities and various models using the same modality.

## 1. Introduction

Neonatal calves possess an immature and naïve immune system and are reliant on the intake of maternal colostrum for the passive transfer of immunoglobulins [1]. Colostrum is the first mammary secretion a cow produces after calving, which serves as an energy source for calves during the first hours of life, stimulates gut function and development, and impacts the calf’s endocrine system [2,3]. Colostrum is a rich source of nutrients, containing 1.85 times more dry matter, 4.52 times more protein, 1.68 times more fat content, and higher concentrations of minerals and vitamins compared to whole milk [4]. In addition, colostrum has a high content of immunoglobulins, which provide immunity to a calf for the first weeks of life. Calves can absorb immunoglobulins from maternal colostrum through their small intestine at birth. However, the closure of intestinal permeability to these proteins accelerates as the calf age exceeds 12 h, with permeability ceasing completely at 24 h postpartum [5]. Bovine colostrum contains a high concentration of immunoglobulin G (IgG), with smaller amounts of immunoglobulin A (IgA) and immunoglobulin M (IgM) [6]. Failure of transfer of passive immunity (FTPI) is a condition in which neonates receive insufficient maternal antibodies [7]. Multiple studies have demonstrated the importance of successful transfer of passive immunity (TPI) for calf survival and health [8,9]. Adequate TPI is associated with short- and long-term health benefits by reducing mortality due to infectious disease and increasing daily gain, feed efficiency, fertility, and milk production in first and second lactations [10]. Failure of transfer of passive immunity is important for both dairy and beef farms because it may increase the risk of health disorders and contribute to antimicrobial use. In addition, the mean total cost per calf with FTPI is estimated at EUR 60 per dairy calf and EUR 80 per beef calf [11].

Several management practices and animal factors, including the timing and amount of colostrum fed, colostrum feeding method, time spent in the maternity area, breed, twin birth, dystocia, dam parity and health status, and herd size are known to be associated with the acquisition of passive immunity in dairy calves [12]. However, risk factors associated with FTPI in beef calves are not well established. Therefore, the main objective of the current study was to summarize the available literature to understand the prevalence of FTPI and the association between FTPI and calf health and identify risk factors for FTPI, as well as define best practices for measuring TPI and diagnosing FTPI in beef calves.

## 2. Materials and Methods

### 2.1. Eligibility Criteria

Eligible studies were those with full texts available in English. Analytic observational and experimental studies were eligible, including controlled trials, cohort studies, case–control studies, and cross-sectional studies. Conference proceedings were included if the abstract had at least 500 words. Book chapters, review articles, case reports, and non-English articles were excluded. We included studies that focused on validating or comparing diagnostic methods for TPI in beef calves. Studies using beef or dual-purpose cattle were included; those using only dairy breeds or calves born on dairies were excluded. Studies evaluating only colostrum quality without also studying the transfer of passive immunity in calves were also excluded.

### 2.2. Search Strategy

The literature was searched through the CAB Direct (via CABI), Scopus, and PubMed databases. Publications from 2003 onwards, i.e., not more than 20 years old at the time of the initial search, were eligible. Electronic databases were searched during the period from May to July 2023. Search terms were developed related to the study population (beef calves), the intervention (colostrum and vaccination), and outcome (passive immunity). The search string was adjusted for each database (Tables S1–S3). The resulting publications were exported, stored, and de-duplicated in Systematic Review Accelerator [13]. Duplicates were removed by matching the first author, title, and publication year. All screening processes were performed using Covidence systematic review software (Veritas Health Innovation, Melbourne, Australia). The search was repeated in January 2025 to include studies that were published after the first search was performed.

### 2.3. Screening Processes

The screening process included two stages: title and abstract screening, and full-text screening. At both stages, 2 reviewers (GM and EA) independently reviewed the articles. When reviewers had conflicting answers, they resolved the conflict through discussion.

Title and abstract screening were performed by looking for the following keywords: beef calves, passive immunity, calving, prepartum vaccination, health, tube feeding, esophageal feeding, and adequate nutrition. Articles that included the following keywords in their title or abstracts were excluded: dairy calves, cows (if no calf), postweaned calves, behavior, and veal calves.

Studies were moved to the full text screen if they included neonatal beef calves and at least one of the other terms. Studies categorized as “yes” or “maybe” were retrieved and included in the full-text screening.

The full-text screening was conducted for all studies that passed the title and abstract screening process. The exclusion criteria were studies in non-beef operations (e.g., veal, dairy), studies on post-weaned beef calves, studies on behavior, and studies conducted on Holstein or other dairy breeds. All studies were categorized as either “include” or “exclude”, and studies that did not fit the inclusion criteria were removed. Those that fit inclusion criteria were eligible for data extraction. During the full-text screening, each study was tagged with either prevalence estimates of FTPI; association between FTPI and calf health; a colostrum management-, calf-, or dam-related factor to describe the risk factor associated with FTPI; or methods of FTPI detection.

### 2.4. Data Extraction

All included studies were extracted from Covidence software into a CSV file and complemented with data manually extracted from articles (Table S4). Data extracted from the articles included the authors, title, year of publication, journal name, study design, risk factor or intervention that was assessed, country of study location, and number of animals in the study. Descriptive statistics were generated in R Studio software (version 2024.12.1), and the numbers of studies and their characteristics were summarized in tables and figures.

## 3. Results

### 3.1. Descriptive Summaries

**Figure 1:**
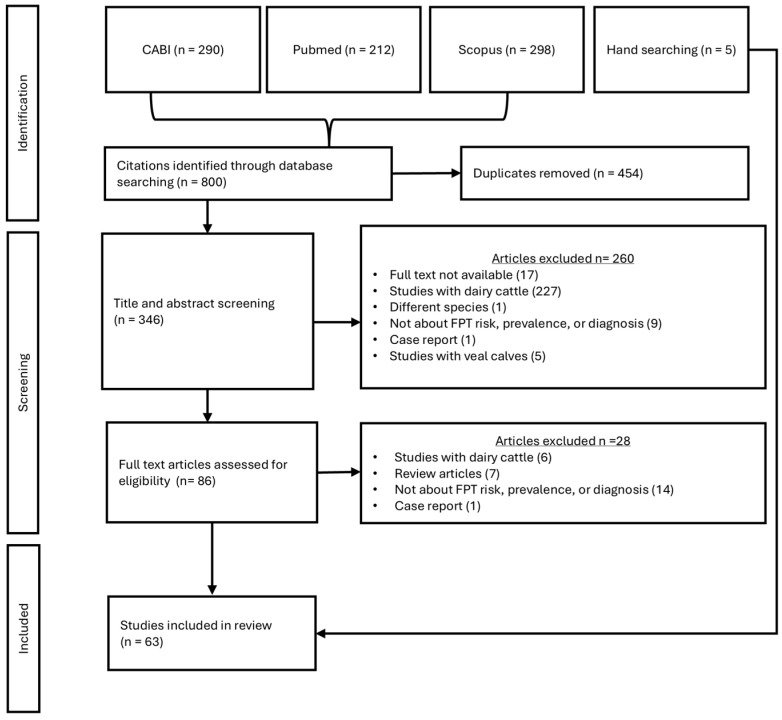
Flow chart of studies published between 2003 and 2025 identified in a database search and reasons for their exclusion from a literature review on risk factors for failure of transfer of passive immunity in neonatal beef calves.

**Figure 2:**
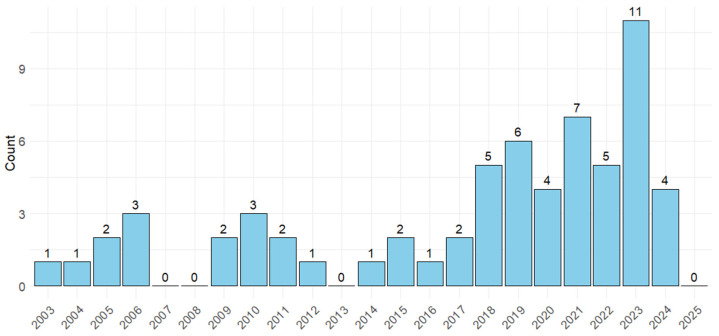
The number of studies that were conducted to identify prevalence of failure of transfer of passive immunity (FTPI), risk factors associated with FTPI in beef calves or the association between FTPI and health outcomes in neonatal beef calves, or the diagnosis of FTPI published each year from 2003 to 2025.

A comprehensive search of electronic databases yielded a total of 800 studies. Following the removal of 454 duplicates, 346 studies were included for initial screening. Subsequently, 260 studies were excluded at the title and abstract stage. The remaining 86 studies underwent full-text screening, with 28 studies being excluded because they were on dairy cattle; were review articles; the focus was not on risk factors for FTPI or the prevalence or diagnosis of FTPI in beef calves; or they were case reports. Ultimately, 63 studies were deemed eligible for data extraction (Figure 1). Most studies were published between 2014 and 2024 (76%; *n* = 48) (Figure 2).

Studies were categorized into each risk factor or intervention investigated, as shown in Table 1.

The majority of studies were conducted in the United States or Canada (*n* = 30), followed by Europe (*n* = 15). One study each was conducted in Australia, New Zealand, and South America (Brazil), and two studies were carried out in Asia (Korea and Turkey) (Table 2). Study design types and breed types mentioned in studies are also listed in Table 2. Of those studies that indicated a breed type, crossbred was the most common type of breed description used (*n* = 31), including the beef–dairy crosses Angus–Holstein (*n* = 1), Angus–Jersey (*n* = 1), Angus–Kiwicross (*n* = 1), Limousin–Friesian (*n* = 2), and the beef cross Charolais–Limousin (*n* = 1). Some studies either did not mention the type of breed (*n* = 8), listed “Other” as breed type (*n* = 1), or said that 31 breed types were represented (*n* = 1). Studies where no specific breed type was mentioned were typically large observational studies. The number of beef calves, dams, or pairs used in studies ranged from 10 to 1568 (median: 84, IQR: 156).

### 3.2. Prevalence Estimates of FTPI in Beef Calves

Five studies reported estimates for the prevalence of FTPI ranging from 5.8% to 34.5% in study calves; however, definitions for FTPI were not consistent. A cross-sectional study of 1131 calves on 84 farms in Great Britain found 15% of calves with serum IgG concentrations < 10g/L and 37% < 24 g/L [9]. In an observational study of 225 calves from 45 cow-calf herds in Quebec, 19% had FTPI based on <10 g/L serum IgG_1_ [14]. An observational study of 935 beef calves from 152 herds in Alberta and Saskatchewan found 5.8% to have FTPI based on serum IgG ≤ 8 g/L [15]. In a population of Irish beef herds, 22% of 82 sampled calves had FTPI based on a zinc sulfate turbidity (ZST) test result <20 units [16]. Also based on a cutoff of 10 g/L serum IgG, 34.5% of 202 Belgian Blue beef calves from the same herd were found to have FTPI [17].

### 3.3. Association Between FTPI and Calf Health Outcomes

Results from studies that have found associations between FTPI and health outcomes, and the cutoffs they determined for various health outcomes are summarized in Table 3. Despite the known importance of passive transfer of immunity to calf health outcomes, not all studies found associations between various measures of TPI and morbidity or mortality in calves [14,15,18]. However, the study by Waldner and Rosengren found associations between IgG serum concentrations below 24 g/L and negative health outcomes, including calfhood treatments with pharmaceuticals other than vaccines for any reason and mortality [15]. Likewise, Bragg et al. found that calves with higher serum IgG levels are at a reduced odds of death or requiring treatment (OR 0.97, 95% CI 0.95–0.99) [19]. In a cohort study of 1568 beef calves, those with FTPI based on a serum IgG1 concentration ≤ 8 g/L were more likely to have a preweaning morbidity event, compared with calves that had adequate passive transfer with a serum IgG_1_ concentration > 16 g/L (OR 2.24 (95% CI, 1.52 to 3.29)). The study suggested 2400 mg/dL (24 g/L) serum IgG_1_ as a cutoff for optimal TPI based on likelihood ratios for disease or death before weaning events in this cohort [8]. In a study of 420 calves from six farms in Alberta, Canada [20], calves with serum IgG concentrations < 10 g/L were more likely to receive treatment (OR 7.9, 95% CI 2.7–23.7) or die (OR 18.5, 95% CI 3.7–93.2). This trend was consistent for calves with a serum IgG concentration < 24 g/L, which also had higher odds of dying (OR 10.1, 95% CI 2.6–40.2). Among 355 Charolais calves, serum IgG levels < 10 g/L were associated with higher mortality (*p* < 0.001) and higher IgG_1_ concentrations were associated with fewer health problems [21]. Finally, an observational study in 1392 Irish beef calves determined various diagnostic cutoffs for tests of passive immunity in terms of morbidity and mortality outcomes [22].

### 3.4. Factors Related to Colostrum Management

Multiple studies evaluated the impact of the way calves receive colostrum on serum IgG or on how these practices affect FTPI status or optimal passive transfer. Often, studies have come to conflicting results on the effects of a practice. However, it is critical to understand that the practice itself may not be the causative factor in affecting serum IgG, but rather there may be an underlying factor, such as dystocia, that results in feeding colostrum via bottle or esophageal tube to weak calves that do not suckle on their own, and which may be the true cause of insufficient TPI. This is especially true for observational studies, where the feeding method is not randomly assigned.

#### 3.4.1. Colostrum Quantity or Volume

A randomized controlled trial conducted by Gamsjäger et al. [23] found that the latency of beef calves to stand and nurse from their dam after being fed colostrum was associated with the volume and colostrum feeding type used at first feeding. Calves fed a volume of 1.4 L with 70 g/L IgG (Bovine Dried Colostrum, Calf’s choice Total, Saskatoon Colostrum Company Ltd., Saskatoon, SK, Canada) nursed from their dams earlier (*p* = 0.003) compared to calves fed 1 L with 100 g/L IgG or calves fed 2 L with 100 g/L IgG of an experimental colostrum product, while calf serum IgG concentration was not affected by treatment. All feedings were performed through esophageal tube feeder.

#### 3.4.2. Colostrum Quality (IgG Concentration or Source)

Three studies evaluated colostral IgG content and source on calf serum IgG and had variable outcomes. The Gamsjäger et al. study mentioned above [23] found that feeding 1 L or 2 L of a colostrum supplement with a high IgG concentration (100 g/L) or 1.4 L of a product with a moderate IgG concentration (70 g/L) (Bovine Dried Colostrum, Calf’s choice Total, Saskatoon Colostrum Company Ltd., Saskatoon, SK, Canada) did not result in statistically significantly different calf serum IgG concentrations in beef calves possibly because calves subsequently also consumed colostrum from their dams. Likewise, Chamorro et al. [24], in a randomized controlled trial, compared calves delivered through C-section and fed either a colostrum replacer product (Calf Choice Total Gold, Saskatoon Colostrum Company, Saskatoon, SK, Canada) providing 60 g of IgG per feeding twice, the same feeding treatment and reuniting calves with their dams after the second feeding, or immediately reuniting calves with their dams after delivery. No difference in serum IgG levels was found between treatment groups. On the other hand, Ahmadi et al. [25] evaluated feeding a colostrum replacer product (Headstart, Saskatoon Colostrum, Saskatoon, SK, Canada) containing 60 g IgG versus natural bovine colostrum from Holstein cows containing > 50 g/L IgG to beef calves in a randomized controlled trial and found higher serum IgG levels (14.7 vs. 10.8 ± 0.92 mg/mL on day 2 (*p* < 0.01)) and lower proportions of calves with FTPI in calves fed natural colostrum (15% vs. 50%, *p* < 0.05), but no difference in growth rate, body frame development, and incidence of diarrhea between treatment groups. Similar amounts of approximately 2.4 L of colostrum are reported to have been consumed by calves in the two treatment groups on average; however, it is unclear whether calves were forced to consume any minimal amount. One of the limitations of this study is that it did not clarify whether the observed differences were due to the source of colostrum or the actual quantity of IgG ingested.

#### 3.4.3. Timing of Colostrum Feeding

The timing of first colostrum feeding is a primary factor for the degree of TPI as intestinal enterocytes lose the ability to absorb intact macromolecules with time after birth. We found two articles that assessed the timing of first colostrum feeding on calf passive immunity. Homerosky et al. [26] found calves with failed colostrum consumption by 4 h after birth had lower serum IgG concentrations (*p* = 0.01), had higher odds of not acquiring optimal passive immunity of ≥ 24 g/L serum IgG (OR 6.4, 95% CI: 1.2–34.4), and higher odds of being treated for preweaning diseases (OR 2.8, 95% CI: 1.1–7.4; *p* = 0.03) such as diarrhea and BRD than those that succeeded, in an observational study enrolling calves that were born via assisted or unassisted delivery. In contrast, in a cohort study of Holstein–Friesian and Belgian Blue pairs, calves were bottle-fed a total of 6 L of their dam’s colostrum separated into three 2 L meals at 2 h, 6 h, and 24 h [27]. In both breeds, serum IgG levels increased with increasing age at first feeding. However, the age at first feeding in study calves ranged only between 10 and 150 min, a timeframe that may not result in significant differences in gut closure.

#### 3.4.4. Method of Colostrum Feeding

Eight studies investigated the relationship between the method of colostrum feeding and calf serum IgG concentration and came to a spectrum of conclusions. An observational study of 84 beef farms in the UK found significant associations between any assistance with colostrum feeding compared to calves that nursed on their own and calf serum IgG levels < 24 g/L (lead to dam OR 1.85 (1.11–3.06 95% CI), bottle-/tube-fed dam’s colostrum OR 2.35 (1.29–4.30 95% CI), bottle-/tube-fed artificial colostrum OR 3.78 (1.86–7.70 95% CI)) The authors also found associations between FTPI of < 10 g/L serum IgG and bottle- or tube-feeding the dam’s colostrum (OR 2.66 (1.32–5.36 95% CI) or colostrum replacer (OR 2.34 (1.09–5.02 95% CI)) [9]. Likewise, a cross-sectional study of six Canadian beef farms found that being fed colostrum or colostrum product by bottle or tube was a risk for FTPI (IgG < 10 g/L) or inadequate transfer of immunity (IgG < 24 g/L) to beef calves [20]. Further, in a randomized controlled trial on the effect of meloxicam for calves requiring calving assistance, bottle- or tube-feeding calves were associated with lower serum IgG concentrations compared with nursing [28]. Pisello et al. [29] found that bottle-feeding colostrum is a risk factor linked to lower serum IgG concentrations in calves, as Chianina beef calves that received colostrum through free suckling showed higher serum IgG concentrations (20.76 ± 11.47) compared to those fed colostrum by bottle (12.80 ± 10.48), although statistical significance was set at *p* = 0.10.

Several studies found no difference between various colostrum feeding methods and FTPI status. The study by Gamsjäger et al. mentioned previously [23] found that use of a nipple bottle when feeding a smaller volume (1 L) of colostrum compared to feeding with an esophageal tube feeder resulted in a shorter latency to stand and nurse (*p* = 0.005), but did not result in differences in serum IgG concentration. However, in this study, no calves were included that nursed exclusively. McGee et al. found no differences in serum Ig concentrations between calves fed colostrum via esophageal feeder or via assisted suckling [30]. In an observational study on the effect of embryo transfer and artificial insemination on the method of feeding colostrum among other outcomes, the feeding method (suckling, bottle feeding, or esophageal tube feeding) was not correlated with FTPI as assessed by serum total protein (STP) < 5.0 g/dL, gammaglobulin < 1.0 g/dL or GGT < 100 IU/L [31].

Only one study found an advantage of bottle feeding over suckling. According to Filteau et al. [14], calves fed colostrum with a nipple bottle had significantly lower odds of experiencing FTPI compared to those that relied solely on the dam without any assistance for colostrum intake (OR 0.06, *p* = 0.014).

#### 3.4.5. Microbial Content of Colostrum

In the study conducted by Van Hese et al. [27], the microbial composition of first-milking colostrum was investigated in dairy Holstein–Friesian (HF) and beef Belgian Blue (BB) cows using amplicon sequencing of bacterial genes. The researchers observed significant differences in bacterial diversity between the HF and BB colostrum samples. Additionally, within each breed, several genera were found to vary in abundance between colostrum samples of different quality. Notably, in the HF cows, the bacterial composition of colostrum leading to low serum IgG levels in the calf differed from that of colostrum leading to high serum IgG levels. No such differences were found in Belgian Blue colostrum samples.

### 3.5. Factors Related to Calves

Calf intrinsic factors include calf sex, whether a calf has a twin, a calf’s vigor at birth, whether a calf is born from a breeding that was performed using reproductive technologies such as embryo transfer of artificial insemination, a calf’s birthweight, the month of birth, and a calf’s cortisol or epinephrine blood levels at birth.

#### 3.5.1. Calf Sex or Twin Status

Five studies that evaluated calf sex or twin status as a risk factor for FTPI or serum IgG levels were observational studies. Male calves were found to have lower serum IgG levels than female calves in two studies [9,32]; however, one of the two studies, by Cavirani et al., included dairy and beef breeds, and the effect may have been confounded by dairy management practices [32]. The three studies that did not find an association between serum IgG and calf sex all controlled for calving assistance in their analysis [14,15,28], whereas the study by Bragg et al. [9] constructed separate models for farmer intervention including calving assistance and intrinsic factors including calf sex, and Cavirani et al. [32] only considered breed, gender, and dam parity as risk factors. One study in Charolais cattle found male calves to have higher IgM levels (2.1 mg/mL versus 1.7 mg/mL, *p* = 0.02) than female calves, but found no difference between IgG_1_ levels [21]. The two studies that assessed twin status as a risk factor both found that twin calves are more likely to have lower serum IgG levels than single calves, OR 3.31 (95% CI 1.64–6.71) for serum IgG < 24g/L [9] and *p* = 0.004 for continuous serum IgG levels [15].

#### 3.5.2. Calf Vigor at Birth

Three studies used calf vigor as a risk factor for serum IgG concentration or inadequate transfer of passive immunity in their analyses, and two found low calf vigor to be associated with less desirable outcomes [26,33]. Homerosky et al. [26] found that calves with a weak suckle reflex had 41.6 (95% CI 7.4–787.5) times higher odds of failing to consume colostrum successfully within 4 h, which in turn was a predictor for not acquiring > 24 g/L serum IgG (OR 6.4, 95% CI 1.2–34.4, *p* = 0.02). Pearson et al. found an incomplete tongue withdrawal (*p* = 0.005) and weak suckle reflex (*p* = 0.02) to be associated with decreased IgG concentration in a cross-sectional study on the effects of calving difficulty on passive immunity [33]. In contrast, the same first author found that a weak suckle reflex led to lower odds of acquiring inadequate passive transfer of < 24 g/L IgG (OR 0.5, *p* = 0.05) in a randomized controlled trial studying the effect of a non-steroidal inflammatory drug for beef calves with assisted births [28]. It was speculated that producers’ awareness of the association between suckle reflex and colostrum consumption led to biased treatments of calves in this study.

#### 3.5.3. Month of Birth, Reproductive Technologies Used at Breeding, Calf Cortisol and Epinephrine Concentrations, and Nonsteroidal Anti-Inflammatory Drugs After Difficult Calving

The observational study by Filteau et al. showed differences in health outcomes for calves born in January through March compared to April, but not in their FTPI status (serum IgG_1_ concentration < 10.0 g/L [14]. One observational study compared Brahman calves conceived either through artificial insemination or in vitro embryo production and found no difference in FTPI between groups, although the analysis was not adjusted for other risk factors [31]. Epinephrine levels measured at birth and on multiple days until day 24 of age were positively associated with peak IgM serum concentrations, while peak cortisol levels from samples collected at the same time points were negatively associated with peak IgM and IgG_2_ serum concentrations in the study on temperament in Brahman calves [34]. One randomized controlled trial evaluated TPI in calves that received a dose of meloxicam (Metacam, Boehringer Ingelheim, Ingelheim, Germany) after a difficult calving compared to a control group [35]. No differences were found in odds of FTPI (OR 0.2, *p* = 0.36) or serum IgG concentrations (*p* = 0.18) between groups.

#### 3.5.4. Calf Birthweight

Calf birthweight was significantly associated with calf serum IgG_1_ (*p* = 0.03) and IgM (*p* = 0.04) in Charolais calves, with heavier calves having slightly lower serum IgM levels and presumably lower IgG_1_ levels, although the direction of the latter association is not explicitly stated in the study [21]. In contrast, the study comparing Brahman calves conceived either through artificial insemination or embryo transfer did not find an association between birthweight and FTPI [31]. Likewise, a study in Brahman calves correlating temperament, endocrine, immune, and growth variables did not find any associations between birthweight and immunoglobulin levels status [34].

### 3.6. Factors Related to Dams

#### 3.6.1. Dam Body Condition Score or Udder Conformation

Results from a mix of study designs [14,15,28,36] showed that dam body condition score (BCS) was not significantly associated with calf FTPI. According to Hickson et al. [37], every one point decrease in front teat placement score (more outward pointing teats) improved IgG (*p* = 0.01) and STP (*p* < 0.01) concentrations in calf serum, while this trend was seen only in calf IgG serum concentrations (*p* = 0.04) for rear teats.

#### 3.6.2. Dam Breed

Eight studies explored the effect of dam breed on the passive immunity of their offspring in a mix of observational and experimental study designs. Most studies included a mix of beef and dairy breeds and their crosses, where some differences between measures of TPI were found; however, there was no consistent pattern for beef or dairy breeds or their crosses. Altvater et al. found no difference in FTPI based on a STP < 5.2 g/dL between beef and dairy calves; however, a larger proportion of beef than dairy calves had excellent passive transfer (STP > 6.2 g/dL, *p* < 0.01) [38]. When comparing serum IgG concentrations in calves from beef × beef crosses versus beef × dairy crosses, Brereton et al. found no differences [39]. On the other hand, a comparison of serum IgG between beef and dairy breeds resulted in lower serum IgG concentrations in calves born to Italian Friesian cows than those born to other dairy breeds (Reggiana and Bianca Modenese) or beef breeds (Piemontese or Limousine) (*p* < 0.001) [32]. A comparison between calves born to either Angus or the crosses Angus–Friesian, Angus–Jersey, Angus–Kiwicross dams showed that calves from Angus cows had lower concentrations of serum IgG (*p* = 0.006), STP (*p* = 0.005), and lower GGT activity (*p* < 0.001) than calves born to the Angus cross dams. The calves from Angus dams also had a lower percentage reaching an adequate IgG serum concentration of 1600 mg/dL compared to the calves from Angus–Kiwicross dams [37]. Likewise, a study comparing passive transfer in Charolais and Hereford × Holstein–Friesian or Limousin × Holstein–Friesian cross dams showed that calves from Charolais cows had significantly lower IgG_1_, IgG_2_, IgA, and total Ig levels than calves from the beef cross dams in three experiments also assessing dam nutrition [40]. Murphy et al. [41] compared serum IgG_1_ between calves of the following breeds and crossbreeds: Limousin × Friesian, Limousin × (Limousin ×Friesian), Limousin, Charolais, and Simmental × (Limousin × Friesian). Calves from the Limousin × Friesian cross dams had higher serum IgG_1_ concentrations than those from other dams in the study except calves from the Simmental × (Limousin × Friesian) dams (*p* < 0.01) [41]. In a different experiment studying pre-calving vaccination of dams with the crosses Limousin × Friesian and Charolais × Limousin, Earley et al. found no differences in IgG or ZST between calves from those dams [42]. Finally, no difference in serum IgG concentration in calves from British, Continental, or other beef breeds was found in the observational study by Waldner and Rosengren [15].

#### 3.6.3. Dam Prepartum Vaccination

Seven studies assessed the effect of vaccinating beef dams before calving. We included studies that compared general vaccination strategies for pre-partum dams and vaccination of dams for specific pathogens or with specific products. Pisello et al. [29] found that calves born from dams that received prepartum vaccination against *Escherichia coli*, rotavirus and coronavirus had significantly higher serum IgG concentrations than calves born from dams that did not receive those vaccinations (*p* = 0.01) in a cross-sectional study of Chianina beef calves. On the other hand, in a randomized experiment, calves born from dams that received prepartum vaccination against *Escherichia coli*, rotavirus, and coronavirus had similar STP, and there was no difference in FTPI between groups, although vaccine-specific IgG_1_ titers were higher in the calves from vaccinated dams [43]. Reppert et al. [44] also found similar serum IgG levels in calves from beef heifers receiving either two doses of a killed vaccine for bovine herpesvirus 1 and bovine viral diarrhea virus 1 and 2 (Triangle 10 HB; Boehringer Ingelheim, St. Joseph, MO, USA) or two doses of saline, while vaccine-specific antibodies titers to these pathogens were higher in calves from vaccinated heifers. Vaccination of beef cows at 228–271 days of gestation with the commercial modified-live vaccine Bovi-Shield GOLD FP 5 L5 (Zoetis, Parsippany, NJ, USA) containing antigens for bovine herpes virus 1, bovine viral diarrhea virus types 1 and 2, parainfluenza virus type 3, and bovine respiratory syncytial virus along with five serovars of *Leptospira* resulted in a higher level of serum antibody titers for multiple viral antigens in dams and their offspring than in calves from dams that only received a dose of the vaccine pre-breeding [45]. Four types of *Clostridium botulinum* vaccines induced higher antibody titers for holotoxins C and D in calves from vaccinated dams than in calves from control dams [46]. Gamsjäger et al. found in an observational study that vaccination of dams during pregnancy for *E. coli*, bovine coronavirus, BVDV, and BHV-1 raised IgG concentrations specific to those pathogens in calves. However, for *E. coli*, this was only the case for vaccination of cows but not heifers [47]. Finally, a study on the immunoregulatory bovine granulocyte colony stimulating factor pegbovigrastim (Imrestor; Elanco Animal Health, Greenfield, IN, USA) administered to pregnant cows found no differences in plasma IgG or overall globulins in calves from dams or control cows [48]. However, calves from treated cows had lower plasma GGT (*p* < 0.05) on day 1 of life, and also gained less body weight than calves from control cows.

#### 3.6.4. Dam Parity

Ten studies compared passive immunity in calves from heifers to those of multiparous cows or studied age of the dam as a risk factor for FTPI or other outcomes of passive immunity. Most studies found higher serum IgG concentrations in calves from multiparous cows [15,20,28,30,39,49]. The observational study by Bragg et al. [9] found calves born from heifers to have higher odds of having a serum IgG concentration < 24 g/L compared to calves from cows (OR 1.57 (95% CI 1.05–2.17). The experimental study by Hickson et al. [37] measured differences in serum IgG and STP in calves from cows of various ages; however, they did not measure STP in calves from heifers. STP was higher in calves from three-year-old cows than older cows (*p* < 0.001), while no clear pattern emerged about serum IgG levels. The two remaining observational studies by Cavirani et al. [32] and Filteau et al. [14] did not detect any effect of dam parity on serum IgG or FTPI status of calves.

#### 3.6.5. Dam Prepartum Nutrition

Thirteen studies evaluated the effect of different pre-parturient diets and supplements to beef dams on growth, health, and TPI of their calves. In the study by Noya et al. [50], calves from dams restricted to 65% of nutritional requirements in the first third of gestation had lower ADG in the preweaning period, although no differences in plasma IgG or IgM after colostrum intake were seen. Nutritional restriction of dams during the last 15 days of gestation (straw-only vs. grass silage) resulted in lower serum IgG_1_ and total IgG concentrations in their calves [30]. In contrast, restriction of beef heifer nutrition to 70% metabolizable energy and protein requirements starting at 160 days of gestation until calving resulted in greater serum IgG and IgA concentrations (*p* ≤ 0.03) in their calves, although those calves showed less vigor at birth (*p* = 0.05) and needed longer to stand after birth (*p* = 0.02) [51]. Apperson et al. [52] demonstrated that calves born to cows fed selenium-biofortified alfalfa hay in the last trimester of pregnancy and received colostrum within the first 12 h of age had higher serum ovalbumin (a surrogate protein marker for IgG absorption) concentrations at 24 and 36 h of age compared to control calves, suggesting that selenium supplementation of dams can improve TPI. Wallace et al. [53] reported that feeding selenium-biofortified hay to beef cows during the last 8 to 12 weeks of gestation improved selenium status in cows and their offspring, as well as the IgG_1_ concentrations in colostrum compared to cows receiving control hay. However, calf serum IgG_1_ concentration was not improved in calves from cows receiving the fortified alfalfa hay. Late-gestation supplementation of rumen-protected essential fatty acids (EFA) (Essentiom, Church and Dwight Co., Ewing, NJ, USA) to the diet of beef cows such as safflower or sunflower seeds, or cottonseed meal, increased serum fatty acid concentrations (*p* < 0.001), colostrum IgG, and calf serum IgG (*p* < 0.01) and increased calf growth up to weaning (*p* < 0.01) [54]. Protein supplementation of 1 kg/day for 14 days prepartum tended to increase calf growth in Droughtmaster cattle in a dry tropical environment. Addition of the protein supplementation (*p* = 0.08) or a yeast product (*p* = 0.10) (NaturSafe, Diamond V, Cedar Rapids, IA, USA) tended to increase plasma IgG concentration in calves. Yeast product addition increased STP (*p* = 0.03) and plasma globulin concentration (*p* = 0.05) in calves [55]. Results obtained by Wojtas et al. [56] suggest that addition of soy lecithin as fat additive (powdered soy lecithin 20 g/cow/day) in the diet of beef cows during the last 4 weeks before calving may have a beneficial impact on colostrum composition by increasing linoleic acid in colostrum (*p* = 0.049). However, no difference in serum IgG concentration between calves from dams with or without supplementation was seen. Supplementation with corn-dried distiller’s grains plus soluble (DDGS) at 0.30% of BW, providing protein and energy during late gestation to beef cows, had no effect on plasma IgG or protein concentrations in calves, but resulted in heavier calves at weaning (*p* = 0.04) [57]. Addition of mannan oligosaccharide to winter supplement from late gestation through 30 days of lactation in spring-calving beef cows in the form of Bio-Mos (Bio-Mos; Alltech Inc., Nicholasville, KY, USA) limited the loss of BCS following parturition (*p* = 0.10) and from the start of the study through weaning (*p* = 0.05). However, Bio-Mos supplementation to dams did not improve the TPI to the calf, nor did it improve calf growth performance [58]. Supplementation of 1000 IU of vitamin E (Vitamin E 405 Natural Source, d-α-tocopherylacetate, ADM Alliance Nutrition Inc., Quincy, IL, USA) per day from 6 weeks prepartum until the breeding season to beef cows increased colostrum concentrations of α-tocopherol and increased α-tocopherol concentration in calves at birth, but passive transfer of IgG did not differ in calf serum or cow colostrum due to maternal vitamin E supplementation [59]. Addition of a mineral and vitamin supplement (Purina Wind and Rain Storm All Season 7.5 Complete, Land O’Lakes, Inc., Arden Hills, MN, USA) throughout gestation to the diet of beef heifers resulted in no differences in serum concentrations of IgG, IgM, IgA, and total Ig in their calves [60]. Similarly, compared to a basal diet meeting all nutritional requirements except Cu, Zn, and Mn, the addition of inorganic or methionine chelates of Cu, Zn, and Mn (MINTREX chelated trace minerals, Novus International, Inc.,St. Charles, MO, USA) at 133% of requirements or a combination of inorganic and chelated Cu, Zn, and Mn at 100% of requirements during the last trimester of gestation and until 11 days postpartum in multiparous beef cows did not result in differences in serum IgG, IgA, or IgM, or any production parameters in their calves [61].

#### 3.6.6. Calving Area, Calving Difficulty, and Non-Steroidal Anti-Inflammatory Drugs for Dystocia

The observational study by Filteau et al. [14] evaluated the type of indoor calving area and found calves born in a stanchion stall to have 10.2 times higher odds of FTPI than those born in a stall or pen (95% CI 2.6–39.6). Five studies reported that difficult calving or dystocia is a risk factor for either FTPI [9], lower serum IgG [15,21], inadequate passive immunity of <24g/L serum IgG [33], or failure of colostrum consumption by 4 h after birth [26] in beef calves. In this last study, calves with a weak suckle reflex had 41.6 times greater odds of failed colostrum consumption within 4 h compared to calves with a strong suckle reflex (*p* < 0.0001). Only one study failed to detect the same trend in Brahman calves [31]. One study tested whether giving the non-steroidal anti-inflammatory drug meloxicam to dams that calved through C-section [62] would result in favorable TPI for their calves. A higher number of calves whose dams received meloxicam (Metacam, Boehringer Ingelheim Animal Health, Ingelheim, Germany) had IgG serum concentrations ≥ 15 g/L (OR 3.88, 95% CI 1.01–14.96) than in the control group.

#### 3.6.7. Genetics and Heritability

Four studies explored heritability and whether there are any genetic markers that are associated with passive immunity measures. The genome-wide association study by Johnston et al. [63] found low heritability (0.02–0.10) of passive-immunity-associated traits in beef calves. One SNP, however, reached Bonferroni genome-wide significance for an association with serum IgG concentration. Several SNPs in the analysis for various other passive transfer parameters approached significance. Similarly, Altvater-Hughes et al. [38] found a heritability of 0.14 for colostral IgG and 0.11 for colostral IgM in beef cows. Heritability estimates were also found to be low to moderate for IgG_1_ in colostrum (0.28) or calf serum (0.36) in a study with primiparous Charolais cows and their calves [21]. A study exploring the various haplotypes of a gene (B2M) encoding part of the immunoglobulin Fc receptor called beta-2-microglobulin found that calves that are homozygous for haplotype B2M 2,2 had 10.6 times higher odds of FTPI (95% CI 2.07–54.24) than other haplotypes [7].

### 3.7. Methods of FTPI Detection in Beef Calves

For results from studies comparing methods of assessment of passive immunity, cutoffs, and test performance measures see Table 4. A total of 11 studies [17,64,65,66,67,68,69,70,71,72,73] validated methods to assess passive immunity in beef calves by comparing them to radial immunodiffusion (RID), ELISA, optical refractometers, or the biuret method. Many of these studies evaluated digital refractometers that provide results faster and at lower cost than the reference standard RID. Dunn et al. found that serum IgG levels measured with a commercial ELISA kit (Bio-X Diagnostics, Jemelle, Belgium) correlated well with serum IgG levels measured by RID, but there was an almost twofold difference between the results measured by the two methods [72]. The source of variance in the results of a commercial IgG RID assay (Triple J Farms, Bellingham, WA, USA) was the focus of a study by Thompson et al. [74]. The authors found that lot and plate contributed minimally to the variance of test results, but intra-assay variance was responsible for most of the variability, meaning that the ring diameter of the same sample varied with each repetition. They also found increased variance in results for samples that required dilution because their precipitin rings were greater than the highest standard and they considered the clinical usefulness of the assay to be limited. All studies mentioned in Table 4 found that the methods under investigation were useful in evaluating passive transfer in beef calves. In particular, handheld refractometers or Brix devices offer the advantage of being cheap and user-friendly alternatives to laboratory methods such as ELISA or RID, although certain differences exist. For example, Kreuder et al. found that results from the turbidimetric immunoassay (TI) (MAI Animal Health, Elmwood, WI, USA) under study frequently underestimated IgG concentrations, while STP measured with a digital refractometer (MISCO Digital-Dairy, Solon, OH, USA) had less correlation with RID results but led to fewer misclassifications of passive immunity categories than the TI method [73]. Some methods worked better at one end of the spectrum, e.g., the smart strips (Bio-X Diagnostics, Rochefort, Belgium) described by Delhez et al. performed better for low than high serum IgG concentrations [71], while the capillary electrophoresis (MiniCap Flex Piercing, Sebia, Lisses, France) tested by Sustronck et al. showed better results at higher cutoffs of serum IgG for FTPI [17].

## 4. Discussion

In this scoping review, we attempted to provide a comprehensive overview of all existing literature from the last 20 years concerning risk factors and interventions associated with FTPI in neonatal beef calves. We divided the findings into prevalence estimates of FTPI in beef calves, associations between FTPI and calf health outcomes, factors related to colostrum management, calf- or dam-related factors, as well as methods of FTPI detection. Most studies were conducted during the past ten years, indicating a growing interest in the topic. While almost half of the studies were conducted in North America, there were also a significant number of European countries that contributed studies on the topic, representing a wide mix of breed types. There is a range of study designs, including experimental, observational, and diagnostic accuracy studies, with the latter focusing on ways to measure and detect indicators of passive immunity in beef calves. There was often no consensus among studies exploring the same risk factors on the strength, or even the direction, of the associations.

### 4.1. Prevalence of FTPI and Associations with Health Outcomes

The most accepted cutoff for FTPI in studies on the prevalence of the condition is 10 g/L serum IgG or IgG_1_, with some studies using a cutoff of 8 g/L and prevalence estimates ranging between 5.8% and 34.5% in study herds. However, evidence exists that calves with serum IgG levels below 16 g/L or even 24 g/L are still at an increased risk of death or disease. Therefore, it may be prudent to strive for serum IgG levels above 24 g/L in beef calves rather than merely avoiding FTPI based on the lower thresholds of 8 or 10 g/L IgG concentration. Based on the current literature, a Brix value of approximately 8.4% or lower has been associated with increased odds of morbidity, respiratory disease, and mortality in calves under 6 months of age. For STP, values below 5.8 g/dL (using a digital refractometer) or below 60–61 g/L (via clinical analyzers) were consistently linked with higher disease and mortality risk. These findings indicate that Brix > 8.4% and STP ≥ 5.8 g/dL (or ≥ 60 g/L) are suitable thresholds for adequate TPI. While less specific than RID, these thresholds provide reliable, field-applicable cutoffs for identifying FTPI and predicting calf health outcomes. With only one study assessing cutoffs for ELISA IgG testing, it is difficult to recommend an optimal cutoff value. As authors found increased odds of mortality in calves up to 6 months of age with at least 9 mg/mL IgG, the optimal cutoff is likely higher, but further studies are required. For ZST testing, at least 18 g/L IgG is an appropriate cutoff level, but more data would be desirable for this method as well.

### 4.2. Factors Related to Colostrum Management

While colostrum yield in beef cows is typically lower than in dairy cows, IgG concentrations in colostrum tend to be higher in beef cows [75]. Studies on colostrum feeding type comparing nipple bottle and esophageal feeder or the quantity and quality of colostrum often showed no difference in outcomes of TPI, which is likely because calves subsequently suckled colostrum from their dams, which may have negated any differences in TPI achieved through feeding alone. However, studies that compared assistance provided to calves with colostrum intake versus suckling from the dam generally found assistance to be associated with less desirable outcomes, especially in observational studies. Although feeding with an esophageal feeder may come with some drawbacks as explained below, assistance with colostrum consumption may also be confounded by time- to-feeding in observational studies of calves with low birth vigor. While using an esophageal tube feeder is convenient and speedy, it is important to note that this method does not trigger the esophageal groove reflex during colostrum administration. Consequently, colostrum is deposited into the reticulorumen. Hence, tube feeding may pose a potential drawback as the delayed emptying of colostrum from the forestomaches into the small intestine may lead to reduced absorption of IgG [76]. In dairy calves, TPI is similar when using a nipple bottle or an esophageal tube if feeding > 3 L of colostrum [77]. However, as beef operations are less likely to store or have access to frozen colostrum, and are more likely to use commercial colostrum products for colostrum feeding, they may not feed colostrum in excess of 3 L. When a nipple bottle or nipple bucket is used for colostrum feeding, suckling triggers the esophageal groove reflex. This reflex results in the direct deposition of colostrum into the abomasum, facilitating rapid emptying into the small intestine for absorption. However, calves that will not voluntarily suckle from their dam or a bottle have no other alternative than to be fed via esophageal feeder. Achieving optimal TPI in those calves lacking vigor at birth may present an ongoing challenge to cow-calf producers but attempts should be made to improve TPI in this group of calves.

Beef cows generally produce higher IgG1 concentrations in first-milking colostrum compared to dairy cows (e.g., 113.4 vs. 42.7 mg/mL) [75,78] and may be different in their antibody composition, although there is considerable variability between studies likely due to breed, parity, and nutrition. Most available colostrum products are derived from dairy colostrum, serum, or whey. Whether colostrum products specifically formulated from beef cows may be more beneficial for feeding beef calves than those produced from dairy colostrum or serum was not the topic of any of the studies in this review.

During the last 20 years, only one study [27] examined the bacterial content of colostrum in terms of differences in the microbiome between low- and high-quality colostrum in two beef cattle breeds. Microbes present in colostrum may be associated with TPI in neonatal calves and may affect IgG absorption. Further studies exploring the factors, whether they are genetic, environmental, or due to management, associated with the abundance of various microbial species and their role in colostrum quality and absorption are desirable.

### 4.3. Calf-Related Risk Factors

#### 4.3.1. Calf Sex, Twin Status, Birthweight

Five studies that evaluated calf sex or twin status as a risk factor for FTPI or serum IgG levels were observational studies. Male calves were found to have lower serum IgG levels than female calves in two studies [9,32]; however, one of the two studies, by Cavirani et al. [32] included dairy and beef breeds, and the effect may have been confounded by dairy management practices. The three studies that did not find an association between serum IgG and calf sex all controlled for calving assistance in their analysis [14,15,28], indicating that male sex is only a risk if it is accompanied by high birthweight. The study by Bragg et al. [9] constructed separate models for farmer intervention including calving assistance and intrinsic factors including calf sex, and Cavirani et al. [32] only considered breed, gender, and dam parity as risk factors. One study in Charolais cattle found male calves to have higher IgM levels (2.1 mg/mL versus 1.7 mg/mL, *p* = 0.02) than female calves, but found no difference between IgG_1_ levels [21]. The two studies that assessed twin status as a risk factor both found that twin calves are more likely to have lower serum IgG levels than single calves, OR 3.31 (95% CI 1.64–6.71) for serum IgG < 24g/L [9] and *p* = 0.004 for continuous serum IgG levels [15], emphasizing the need to monitor twin calves for colostrum consumption.

#### 4.3.2. Calf Vigor at Birth and Nonsteroidal Anti-Inflammatory Drugs After Difficult Calving

The relationship between calf vigor and passive transfer of immunity has been explored in several studies, with most findings suggesting that low calf vigor is associated with reduced serum IgG concentrations and increased risk of FTPI. Two of the three reviewed studies identified weak suckle reflex or incomplete tongue withdrawal as significant predictors of lower IgG levels in neonatal calves [26,33]. These findings align with the biological understanding that vigorous, timely colostrum intake is critical for the successful absorption of immunoglobulins during the narrow window of gut permeability post-birth. These results suggest that integrating calf vigor assessments into on-farm colostrum management protocols may support more targeted interventions for at-risk calves. Dystocia is thought to be a painful procedure for calves, and the administration of meloxicam was thought to reduce pain, leading to calves that would stand up faster, and therefore consume colostrum earlier and more frequently in the study by Pearson et al. [35]. Authors did not observe any differences in TPI or indicators of pain between groups or whether calves stood and nursed within an hour but calves in the treatment group gained more weight in the first week of life. Sample size limitations were thought to play a role in the obtained results. Also, due to on-farm protocol, farm staff intervened with colostrum consumption by 1 to 4 h after birth, which may have been confounding indicators of TPI.

#### 4.3.3. Month of Birth

The single study evaluating month of birth as a risk factor [14] found no differences in FTPI between months despite the authors’ hypothesis that cold stress in colder months may lead to calves’ reluctance to stand and suckle voluntarily based on previous evidence [79]. The authors speculate that because most calves in the study were born inside, that effect was blunted. Environmental factors such as weather, however, may affect calf vigor and should be taken into account during calving.

#### 4.3.4. Calf Blood Cortisol and Epinephrine Concentration

The main objective of the study on calf endocrine levels by Burdick et al. [34] was to examine relations among growth, endocrine, immune, and temperament variables in neonatal Brahman calves. Elevated calf blood cortisol levels are expected at birth due to fetal and maternal blood cortisol concentrations in the lead up to, and during, parturition [80] and help neonatal vigor. Despite this fact, the article describes a negative correlation between calf blood cortisol and IgM, which the authors explain by the potential immunosuppressive effect of cortisol. The positive correlation between epinephrine and IgM and IgG_2_ observed in study calves may be explained by a similar effect of this hormone on calf vigor without the immunosuppressive outcomes.

### 4.4. Dam-Related Factors

#### 4.4.1. Dam Breed and Body Condition

The studies that included dam breed as a risk factor for FTPI covered a multitude of different breeds. There are no clear patterns as to breed advantages, as individual differences may be a large contributor to variability, although Angus and Charolais purebred cows had lower colostral IgG concentrations than crossbred cows in two studies [37,40]. Crossbreeding is a common practice in the beef industry to combine desirable traits from multiple breeds [81]. There may be a heterosis advantage for crossbred cattle that could be further evaluated for the major beef breeds and their crosses. It was surprising to see that studies found no association between dam BCS and FTPI. In a previous experimental study, calves from heifers with a BCS of 3 or 4 had lower serum IgG concentrations than those from heifers with a BCS of 5, 6, or 7, while there was no difference in colostral IgG concentration [82]. The authors attribute the lower serum IgG concentrations in calves from heifers with lower BCS to decreased volume of colostrum, or decreased calf vigor. The effect seems to be more pronounced in heifer dams that are still growing, while in older cows, BCS is less of a risk factor for FTPI. Another explanation for the lack of correlation of BCS with FTPI may be limited variability of BCS observed in study animals; however, variability is not always easily assessed from the data provided in studies. For example, Lake et al. [36] conducted a controlled experimental study comparing cows with BCS 4 versus BCS 6, using a 1–9 scale, and found no significant effect of maternal BCS on the passive transfer of immunoglobulins in calves. In contrast, Waldner and Rosengren [15], using the same 1–9 BCS scale, reported that calves born to cows with a BCS < 5 had higher serum IgG concentrations than those from cows with BCS ≥ 5 in a large observational study in western Canada. Filteau et al. [14] used a 1–5 BCS scale, where nearly half of the 209 dams had a BCS between 3 and 3.5, and no cow was classified as overly fat. Their analysis showed no significant association between BCS and FTPI. Similarly, in the study by Pearson et al. [28] also using a 1–5 scale, the average BCS was 3.6 (sd = 0.9). A variable for BCS was tested but not included in their final model for TPI. However, in this randomized controlled trial, confounding by variables such as BCS should have been minimal as it was not their primary predictor.

#### 4.4.2. Calving Difficulty, Calving Area, and Non-Steroidal Anti-Inflammatory Drugs for Dystocia

Based on the study conducted by Homerosky et al. [26], it is recommended that a calf with a weak suckle reflex at birth receives human intervention to ensure timely colostrum consumption, regardless of calving ease, although in extensive outdoor calving environments, this may not always be feasible. Additionally, calves undergoing a difficult birth should likely be closely monitored for colostrum consumption, even those with a strong suckle reflex. Monitoring calves that had a difficult birth and providing timely colostrum to them is likely among the most important measures producers can take to prevent FTPI. Calving in a stanchion stall was associated with FTPI in one study [14]. The authors suggest that this type of stall, where cows are locked up in stanchions, prohibits normal grooming behavior by dams to simulate calves, which may delay the calves’ first attempts to suckle. Allowing interaction between dam and calf after parturition is, therefore, important for TPI, and systems that inhibit it are not recommended. In the study by Guatteo et al. [62], calves born via C-section from cows treated with meloxicam had higher IgG serum concentrations than calves whose dams were not treated with the drug after C-section. Since the colostrum IgG concentrations were comparable between the groups, the authors conclude that the better TPI was due to either earlier or higher colostrum intake by calves. Pain medication for painful procedures such as C-section not only improves animal welfare, but may also lead to beneficial outcomes for health and production.

#### 4.4.3. Dam Parity

The majority of studies in this review that examined parity as a risk factor for FTPI found calves from primiparous cows to have reduced TPI compared to calves from higher lactation cows. The higher immunoglobulin mass found in colostrum of multiparous cows partly explains these results [30]. Furthermore, higher incidence of dystocia in heifers may lead to less vigorous calves at birth or a poor mothering score and consequently longer latency to first suckle, which may compromise TPI [82].

#### 4.4.4. Dam Nutrition

Most studies that focused on improving cow nutrition in late gestation have shown some advantage over control diets in parameters important for cow or calf health and/or reproductive or growth performance. Whether these advantages are of significance to herd health or whether they are economically viable may depend on each situation. For example, feeding selenium biofortified hay may only be of importance if the regular feed source of cattle is deficient in selenium, or where supplementation with other forms of selenium may not be feasible. Economic assessments were rarely included in studies; however, whether a particular feeding management practice is economically viable must also be assessed on a case-by-case basis. Many of the studies evaluated either showed no improvement in colostrum quality with supplementation, or no improved TPI in calves with supplementation despite better colostrum quality in supplemented dams [30,50,51,53,56,57,58,59,60,61]. A few studies showed improvements in TPI parameters [52,54,55]. It is difficult to compare studies with different outcomes, different ways to measure the outcomes, and herd and environmental differences between studies. The benefits of fetal programming are not evaluated or measured in this review; however, dam nutrition seems to play a minor role in the assurance of TPI in beef calves despite a large body of available studies. It was surprising to see that nutrient restrictions during late pregnancy of dams resulted in no differences in TPI in their calves in some studies [50,51]. A review by McGee and Early on passive immunity in beef-suckler calves confirms this trend in the studies they evaluated [75]. A possible explanation of this trend is the greater immunoglobulin concentration in the colostrum of nutrient-restricted dams allowing the calf to absorb high amounts of antibodies during initial suckling events. Another physiological mechanism for the effect suggested by the same authors is the delayed maturation of small intestines of calves from nutrient-restricted dams, leading to higher absorption of immunoglobulins in neonates [51]. However, the long-term disadvantages on health and production parameters in calves of nutrient-restricted dams may outweigh any short-term benefits.

#### 4.4.5. Dam Prepartum Vaccination

All studies on dam prepartum vaccination were able to provide evidence for improved TPI from dams to calves through colostrum consumption. Recent studies by Gamsjäger et al. [20,47] found that higher *E. coli-*, BRoV-, BVDV-, PI-3-, and BHV-1-specific IgG concentrations by vaccinating dams in the pre-calving period were significantly associated with lower odds for pre-weaning treatment. Therefore, prepartum vaccination of beef cows could be used as a preventive strategy to decrease the occurrence of respiratory and digestive diseases in neonatal beef calves, especially early in life, when the calf’s own immune system is still underdeveloped. Some authors monitored the decline in maternal antibodies in calves and based calf vaccination recommendations on those observations. We only evaluated the studies published since 2003, but a vast body of literature was published on the topic prior to our cutoff date, and we refer to these studies on specific vaccines or diseases.

#### 4.4.6. Genetics and Heritability

Heritability estimates for FTPI in beef breeds were low to moderate in the studies evaluated in this review. Nevertheless, genetic markers have been identified [7,63] that are associated with FTPI and further research in this area may be warranted. It remains to be seen whether selection for passive immunity will be more valuable than proper herd health measures in preventing calves with FTPI.

### 4.5. Methodology to Assess FTPI in Beef Calves

A variety of methods were evaluated in studies on determining cutoffs for FTPI in beef calves. Table 4 provides a reference to compare various methods to a reference standard, mostly RID including measures of accuracy. For herd health purposes, digital or optical refractometers may provide a viable alternative to more time and resource demanding laboratory methods. New improved technology will likely continue to become available in the future and further studies should be consulted for updates.

The most recent and commonly used tools for detection of passive immunity in calves are Brix refractometers, which approximate the percentage of total solids (% Brix) in fluids such as calf serum. Brix refractometers are available in optical or digital versions, with the latter being more attractive as it provides a quick and objective measurement of the Brix percentage in the sample.

One study assessed the effectiveness of an immunochromatographic assay kit called SmartStrips, developed by Bio-X Diagnostics, for detecting passive immunity [71]. These assays are conducted using whole blood for practical convenience, eliminating the need to wait and centrifuge blood to obtain serum. Despite being run on whole blood, the results are expressed in serum IgG concentrations (mg/mL). The test principle is the competition between bovine IgG in the sample and bovine IgG coated on the test line for the gold-labeled mobile antibodies specific to bovine IgG. The SmartStrips immunochromatographic assay can identify calves with poor TPI (serum IgG < 10 mg/mL).

## 5. Conclusions

Transfer of passive immunity is clearly a multifactorial process with significant individual variability that cannot be easily attributed to specific factors in isolation. Over the past decade, there has been growing interest in identifying risk factors for failure of transfer of passive immunity (FTPI) and establishing best management practices to ensure optimal TPI, a trend that continues to provide valuable insights for veterinarians and producers. Serum IgG concentrations above 16 mg/mL have been suggested as indicative of adequate passive transfer in beef calves, while a threshold of 24 mg/mL is increasingly accepted as optimal, based on studies linking it to improved health outcomes. Effective TPI in beef calves is likely influenced most by good breeding and calving management, assessment of newborn vigor, and overall herd health practices, including vaccination programs. Since FTPI remains a major contributor to morbidity and mortality in young calves, increasing producer awareness of methods to enhance passive immunity and reduce known risk factors may lead to better calf health and performance.

## 6. Future Directions

Further research is warranted on colostrum replacer products derived from beef cows as their antibody profile and colostrum composition may differ from those of dairy cows that are the source of currently marketed colostrum-replacer products and to meet the needs of beef ranchers. Additional studies on the microbiome of beef colostrum could result in a better understanding of the microbial composition in various qualities of colostrum. More targeted studies trying to unravel how crosses of the major beef breeds compare to purebred cattle in terms of their colostrum quality may be helpful for breeding decisions in commercial herds along with further studies on any genetic markers for colostrum quality. Finally, the inclusion of breeding management as a risk factor for FTPI may be helpful in further solidifying the association between dystocia and FTPI.

## Figures and Tables

**Table 1:** Number of extracted studies and their characteristics as risk factors for failure of transfer of passive immunity (FTPI), associations between FTPI and health outcomes, methods of FTPI detection, or prevalence estimates of FTPI in beef calves from 2003 to 2025. A study may be counted in more than one category.

Topics, Risk Factors, or Interventions	Number of Studies
Prevalence estimates of FTPI in beef calves	5
Association between FTPI and calf health outcomes	8
Factors related to colostrum management	
Colostrum quantity or volume fed	1
Colostrum quality (IgG concentration or source)	3
Timing of colostrum feeding	2
Colostrum microbial content	1
Colostrum feeding method	8
Factors related to calves	
Calf sex or twin status	6
Calf vigor at birth	3
Reproductive technologies used during breeding	1
Calf birth weight	3
Month of birth	1
Calf cortisol and epinephrine concentrations	1
Nonsteroidal anti-inflammatory drugs after difficult calving	1
Factors related to dams	
Dam body condition score (BCS) or udder conformation	5
Dam breed	8
Dam prepartum vaccination	7
Dam parity	10
Dam prepartum nutrition	13
Calving area (type and location)	1
Calving difficulty	8
Genetics and heritability	4
Nonsteroidal anti-inflammatory drugs prior to C-section	1
Methods of FTPI detection in beef calves	11

**Table 2:** Number of extracted studies and countries where studies were performed, and study design used, as well as breed types of cattle used in experiments in a scoping review of studies on FTPI in beef calves published from 2003 to 2025. For the breed category, a study may be counted in more than one breed type.

Country Where Study Was Conducted	*n*
USA	18
Canada	12
Ireland	9
Brazil	5
Belgium	4
France	3
Italy	4
Great Britain	2
Australia	1
Korea	1
New Zealand	1
Poland	1
Spain	1
Turkey	1
Study design	*n*
Randomized controlled trial	27
Cohort study	13
Diagnostic accuracy study	11
Cross-sectional study	11
Case–control study	1
Breed	*n*
Crossbred	31
Angus	10
Charolais	8
Belgian Blue	5
Limousin	5
Simmental	4
Hereford	3
Brahman	2
Chianina	2
Nelore	2
Aberdeen	1
Aubrac	1
Droughtmaster	1
Hanwoo	1
Padra de Montana	1
Piemontese	1
Pirenaica	1
Salers	1

**Table 3:** Cutoffs for diagnostic assays for indicators of passive immunity in beef calves and outcomes assessed in studies published between 2003 and 2025.

Method	References	Cutoff	Outcome Effect Measure (95% CI)
Serum IgG RID	Filteau et al. [14]	<10.0 g/L	No association between FTPI and health status (*p* = 0.17) in calves 24 h to 7 d old
	Waldner and Rosengren [15]	<8 g/L <16 g/L	No association between FTPI and calf death or treatment (*p* > 0.25)
		<24 g/L	Calf death before 3 months of age; OR 1.6 (1.1–2.3) Calf treatment for any reason; OR 1.5 (1.0–2.3)
	Bragg et al. [19]	Every g/L increase	Death and/or treatment for disease within 9 months OR 0.97 (0.95–0.99)
	Dewell et al. [8]	<2400 mg/dL	Disease before weaning Likelihood ratio 1.6 (1.19–2.28) Death before weaning: Likelihood ratio 2.7 (1.34–5.36)
		≥2700 mg/dL	3.4 kg higher body weight at 205 days
	Gamsjäger et al. [20]	<10.0 g/L	Treatment for disease OR 7.9 (2.7–23.7) Mortality OR 18.5 (3.7–93.4)
		<24.0 g/L	Mortality OR 10.1 (2.6–40.2)
	Martin et al. [21]	<10 g/L	Mortality Chi-squared test compared to calves with IgG levels > 20 g/L, *p* < 0.001
STP	Perrot et al. [18]	<5.1 g/dL	No association between prevalence of omphalitis and FTPI (*p* = 0.63)
	Todd et al. [22] digital refractometer	<5.8 g/dL	Morbidity due to any cause (0–6 months) OR 1.6 (1.1–2.3)
		<5.8 g/dL	BRD (0–6 months) OR 2.3 (1.2–4.3)
		<6.3 g/dL	Other causes of disease (0–3 months) OR 2.5 (1.2–5.3)
		<5.3 g/dL	Mortality (0–6 months) OR 3.9 (2.0–7.7)
	Todd et al. [22] clinical analyzer	<61 g/L	Morbidity due to any cause (0–3 months) OR 1.5 (1.1–2.2)
		<56 g/L	BRD (0–1 months) OR 6.2 (1.7–22.6)
		<61 g/L	Other causes of disease (0–6 months) OR 2.1 (1.2–3.7)
		60 g/L	Mortality (0–6 months) OR 4.3 (1.8–10.1)
Serum IgG ELISA	Todd et al. [22]	<8 mg/mL	Morbidity due to any cause (0–3 months) OR 2.0 (1.3–2.9)
		<8 mg/mL	BRD (0–1 months) OR 4.5 (1.4–14.5)
		<8 mg/mL	Other causes of disease (0–1 months) OR 1.8 (1.0–3.1)
		<9 mg/mL	Mortality (0–6 months) OR 2.8 (1.4–5.8)
Serum total solids percentage, Brix	Perrot et al. [18]	<8.1%	No association between prevalence of omphalitis and FTPI (*p* = 0.86)
	Todd et al. [22]	8.4%	Morbidity due to any cause (0–6 months) OR 1.5 (1.1–2.2)
		8.4%	BRD (0–1 months) OR 7.2 (1.8–30.0)
		8.4%	Other causes of disease (0–6 months) OR 1.7 (1.1–2.9)
		8.4%	Mortality (0–6 months) OR 2.8 (1.4–5.6)
Serum IgG globulin, clinical analyzer	Todd et al. [22]	26 g/L	Morbidity due to any cause (0–3 months) OR 1.6 (1.1–2.4)
		32 g/L	BRD (0–1 months) OR 6.3 (1.3–29.8)
		40 g/L	Other causes of disease (0–1 months) OR 3.1 (1.2–8.0)
		32 g/L	Mortality (0–6 months) OR 3.4 (1.5–7.5)
Serum IgG Zinc sulfate turbidity, units	Todd et al. [22]	12 g/L	Morbidity due to any cause (0–3 months) OR 1.8 (1.3–2.6)
		14 g/L	BRD (0–1 months) OR 11.2 (2.1–60.4)
		18 g/L	Other causes of disease (0–1 months) OR 2.2 (1.1–4.3)
		14 g/L	Mortality (0–6 months) OR 3.4 (1.6–7.0)

**Table 4:** Comparison of various diagnostic methods to detect FTPI in beef calves and in studies published between 2003 and 2025.

Reference	Reference Method and Cutoff	Comparative Method and Cutoff	Measures of Test Performance
Dunn et al. [72]	RID serum IgG concentration	Commercial serum ELISA	R^2^ = 0.97, *p* < 0.001 Fixed bias (sRID–ELISA) = 12.36 ± 6.60 mg/mL
		Zinc sulfate turbidity	R^2^ = 078, *p* < 0.001
Akköse et al. [64]	RID serum IgG concentration	Digital Brix refractometer	
	<10 mg/mL	<8.5%	Se 100% (95% CI 87.9–100), Sp 94.2% (95% CI 89.6–97.2)
	<16 mg/mL	<8.5%	Se 92.1% (95% CI 78.6–98.2) Sp 97.6% (95% CI 93.9–99.3)
	<24 mg/mL	<10.1%	Se 88.8% (95% CI 79.7–94.7) Sp 67.2% (95% CI 58.1–75.4)
		Digital STP refractometer	
	<10 mg/mL	<5.2 g/dL	Se 100% (95% CI 87.9–100), Sp 93.6% (95% CI 88.9–96.8)
	<16 mg/mL	<5.2 g/dL	Se 92.1% (95% CI 78.6–98.2) Sp 97.0% (95% CI 93.0–99.0)
	<24 mg/mL	<6.4 g/dL	Se 87.5% (95% CI 78.2–93.8) Sp 69.7% (95% CI 60.7–77.7)
Delhez et al. [71]	Bovine IgG ELISA	Immunochromatographic assay for serum IgG with EDTA blood	
	<10 mg/mL	<10 mg/mL	Se 83% Pr 94%
	10.0–14.9 mg/mL	10.0–14.9 mg/mL	Se 78% Pr 58%
	15.0–19.9 mg/mL	15.0–19.9 mg/mL	Se 50% Pr 86%
	>20.0 mg/mL	>20.0 mg/mL	Se 100% Pr 70%
		Immunochromatographic assay for serum IgG with heparin blood	
	<10 mg/mL	<10 mg/mL	Se 96% Pr 81%
	10.0–14.9 mg/mL	10.0–14.9 mg/mL	Se 72% Pr 90%
	15.0–19.9 mg/mL	15.0–19.9 mg/mL	Se 50% Pr 43%
	>20.0 mg/mL	>20.0 mg/mL	Se 74% Pr 70%
Drikic et al. [69]	RID serum IgG concentration	Split trehalase IgG assay	
	24 mg/mL	OD 450 nm 0.3	Se 69.2% Sp 97.2%
Gamsjäger et al. [65]	RID serum IgG	Digital Brix refractometer	
	<10 g/L	≤7.9%	Se 81.2% (95% CI 54.4–96.0) Sp 94.8% (95% CI 92.0–96.8)
	<16 g/L	≤8.3%	Se 88.2% (95% CI 72.5–96.7) Sp 90.9% (95% CI 87.5–93.7)
	<24 g/L	≤8.7%	Se 80.0% (95% CI 68.7–88.6) Sp 93.0% (95% CI 89.6–95.5)
		Digital and optical STP refractometers	
	<10 g/L	≤5.1 g/dL	Digital Se 100% (95% CI 79.4–100) Sp 91.4% (95% CI 88.1–94.0) Optical Se 100% (95% CI 79.4–100) Sp 93.7% (95% CI 90.8–95.9)
	<16 g/L	≤5.1 g/dL	Digital: Se 94.1% (95% CI 80.3–99.3) Sp: 95.3% (95% CI 92.6–97.3) Optical: Se 85.3 (95% CI 68.9–95.0) Sp 97.0 (95% CI 94.7–98.5)
	<24 g/L	≤5.7 g/dL	Digital: Se 95.7 (95% CI 88.0–99.1) Sp: 93.3 (95% CI 90.0–95.7) Optical: Se 91.4 (95% CI 82.3–96.8) Sp 91.2 (95% CI 87.5–94.0)
Kreuder et al., 2022 [73]	RID serum IgG	Turbidimetric immunoassay	
	<18.0 g/L	9.89 g/L	Se: 0.910 (95% CI 0.861–0.951) Sp: 0.888 (95% CI: 0.772–1)
	<25.0 g/L	13.76 g/L	Se: 0.813 (95% CI 0.729–0.885) SP: 0.818 (95% CI 0.712–0.909)
		Digital STP refractometer	
	<18.0 g/L	5.5 g/dL	Se: 0.818 (95% CI 0.761–0.869) Sp: 0.75 (95% CI 0.55–0.9)
	<25.0 g/L	6.0 g/dL	Se: 0.756 (95% CI 0.677–0.827) Sp: 0.754 (95% CI 0.652–0.855)
		Serum gamma-glutamyl transferase	
	<18.0 g/L	2303 IU/L	Se: 0.737 (95% CI 0.669–0.8) Sp: 0.7 (95% CI 0.5–0.9)
	<25.0 g/L	1831 IU/L	Se: 0.905 (95% CI 0.849–0.952) Sp: 0.406 (95% CI 0.290–0.522)
Pisello et al., 2021 [66]	RID serum IgG	Digital STP refractometer	
	<16 g/L	51 g/L	Se 63% Sp 96%
		Optical STP refractometer	
	<16 g/L	52 g/L	Se 69% Sp 90%
		Digital Brix refractometer	
	<16 g/L	8.3%	Se 77% Sp 92%
		Optical Brix refractometer	
	<16 g/L	8.3%	Se 66% Sp 92%
De Souza et al., 2015 [68]	STP optical refractometer	STP digital refractometer	No specific values mentioned, Pearson correlation between method results = 0.9588
Sustronck et al., 2022 [17]	RID serum IgG	Digital Brix refractometer	
	10 g/L	8.4%	Se 80.9 (95% CI 67.6–91.3) Sp 89.5 (95% CI 81.9–96.5)
	15 g/L	8.9%	Se 77.9 (95% CI 69.0–86.0) Sp 90.2 (95% CI 78.7–97.7)
	20 g/L	9.4%	Se 89.6 (95% CI 84.3–94.1) Sp 88.3 (95% CI 68.5–98.4)
		Serum protein capillary electrophoresis	
	10 g/L	10 g/L	Se 81.8% (95% CI 68.0–92.5) Sp 91.0% (95% CI 83.5–97.7)
	15 g/L	15 g/L	Se 92.4% (95% CI 85.4–97.6) Sp 80.0% (95% CI 65.6–91.5)
	20 g/L	20 g/L	Se 98.3% (95% CI 95.1–99.9) Sp 87.6% (95% CI 63.0–98.7)
Vandeputte et al., 2011 [67]	Serum IgG with biuret method	STP handheld refractometer with automatic temperature compensation (ATC), Atago conversion	
	1600 mg/dL	58 g/L	Se 100% (95% CI 82–100) Sp 90% (95% CI 82.1–94.6)
		STP handheld refractometer with ATC, Wolf conversion	
	1600 mg/dL	54 g/L	Se 100% (95% CI 82–100) Sp 93.3% (95% CI 86.2–96.9)
		STP standard laboratory refractometer without ATC, Atago conversion	
	1600 mg/dL	56 g/L	Se 100% (95% CI 82–100) Sp 91.1% (95% CI 83.4–95.4)
		STP digital ATC handheld	
	1600 mg/dL	56 g/L	Se 100 (95% CI 82–100) Sp 92.2 (95% CI 84.8–96.2)

Abbreviations: RID—radial immunodiffusion, Se—sensitivity, Sp—specificity, Pr—precision, Acc: accuracy.

## Data Availability

The search strings for the databases utilized in the literature search are provided in the Supplementary Materials. A list of articles included in the scoping review is also available as Supplementary Materials.

## Supplementary Materials

The following supporting information can be downloaded at: https://www.mdpi.com/article/10.3390/ani15142072/s1, Table S1: Search string for CAB Direct (via CABI) database. Search terms were developed with a combination of terms related to the study population, intervention (colostrum, vaccine) and outcome (passive immunity); Table S2: Search string for PubMed database. Search terms were developed with a combination of terms related to the study population, colostrum, and the other outcomes; Table S3: Search string for Scopus database. Search terms were developed with a combination of terms related to the study population, colostrum, and the other outcomes. Table S4: List of articles in scoping review including authors, title, year of publication, journal, doi, topic for inclusion, study design, country of study location, breeds of cattle in study, and number of cattle in study.
