# Supplementary material for: Failure of Passive Immune Transfer in Neonatal Beef Calves: A Scoping Review
Source: Animals (Basel). 2025 Jul 14;15(14):2072. doi: 10.3390/ani15142072 (PMC12291800; doi:10.3390/ani15142072)
Supplement: Supplementary file 1 [file animals-15-02072-s001.zip › Table S1 CAB search.pdf]

Table S1: Search string for CAB Direct (via CABI) database. for a scoping review on failure of passive immune transfer in neonatal beef calves. Search terms were developed with a combination of terms related to the study population, the interventions colostrum and vaccination, and the outcome passive immunity, as well as a filter for publication year.

CAB 429 results (search was repeated in January 2025 yielding additional results)

**Theme search**	**Synonyms (keywords)**
**#1 calves**	**de:(calves OR "**calf feeding") **OR ti:(calf OR calves OR calf-cow OR "newborn beef" OR "cow-calf" OR (neonat* NEAR/2 calves)) OR ab:(calf OR calves OR calf-cow OR "newborn beef" OR cow-calf OR (neonat* NEAR/2 calves))**
**#2 colostrum**	**de: ("colostrum" OR "immunoglobulins" OR "IgG") OR id:("gamma-globulins") OR ti:("colostrum" OR "IgG" OR "immunoglobulins G" OR "immune globulins G" OR suckling OR beef-suckler) OR ab:("colostrum" OR "IgG" OR "immunoglobulins G" OR "immune globulins G" OR suckling OR beef-suckler)**
**#3 passive immunity**	ti:("passive immun*" OR "passive transfer") OR ab:("passive immun*" OR "passive transfer") OR de:("passive immunity" OR "passive immunization" OR "colostral immunity")
**#4 beef**	**Title: (beef OR veal) OR title:(Angus OR Ayrshire OR Boran OR Brahman OR Brangus OR Braunvieh OR Charolais OR Fleckvieh OR Friesian OR Gelbvieh OR Gir OR Hereford OR Holstein OR Jersey OR Limousin OR Longhorn OR Nellore OR Ongole OR Sahiwal OR Sanga OR Shorthorn OR Simmental OR Wagyu) OR ab:(beef OR veal) OR ab:(Angus OR Ayrshire OR Boran OR Brahman OR Brangus OR Braunvieh OR Charolais OR Fleckvieh OR Friesian OR Gelbvieh OR Gir OR Hereford OR Holstein OR Jersey OR Limousin OR Longhorn OR Nellore OR Ongole OR Sahiwal OR Sanga OR Shorthorn OR Simmental OR Wagyu) OR de:("**beef cattle")
**#5 vaccine**	**ti:("neonatal vaccin*" OR "beef calf vaccin*" OR "prepartum vaccin*") OR ab:("neonatal vaccin*" OR "beef calf vaccin*" OR "prepartum vaccin*")**
**#6**	**#5 OR #2**
**#7**	**#6 AND #4 AND #3 AND #1**<br><br>**(Resulted in 430 documents)**
**#8**	**#7 AND yr:[2003 TO 2023] AND (language:("English") (NOT (item-type:(("Book chapter")**

	(Resulted in 238 documents)
**Copy and paste**	(((ti:("neonatal vaccin*" OR "beef calf vaccin*" OR "prepartum vaccin*") OR ab:("neonatal vaccin*" OR "beef calf vaccin*" OR "prepartum vaccin*")) OR (de: ("colostrum" OR "immunoglobulins" OR "IgG") OR id:("gamma-globulins") OR ti:("colostrum" OR "IgG" OR "immunoglobulins G" OR "immune globulins G" OR suckling OR beef-suckler) OR ab:("colostrum" OR "IgG" OR "immunoglobulins G" OR "immune globulins G" OR suckling OR beef-suckler))) AND (Title: (beef OR veal) OR title:(Angus OR Ayrshire OR Boran OR Brahman OR Brangus OR Braunvieh OR Charolais OR Fleckvieh OR Friesian OR Gelbvieh OR Gir OR Hereford OR Holstein OR Jersey OR Limousin OR Longhorn OR Nellore OR Ongole OR Sahiwal OR Sanga OR Shorthorn OR Simmental OR Wagyu) OR ab:(beef OR veal) OR ab:(Angus OR Ayrshire OR Boran OR Brahman OR Brangus OR Braunvieh OR Charolais OR Fleckvieh OR Friesian OR Gelbvieh OR Gir OR Hereford OR Holstein OR Jersey OR Limousin OR Longhorn OR Nellore OR Ongole OR Sahiwal OR Sanga OR Shorthorn OR Simmental OR Wagyu) OR de:("beef cattle")) AND (ti:("passive immun*" OR "passive transfer") OR ab:("passive immun*" OR "passive transfer") OR de:("passive immunity" OR "passive immunization" OR "colostral immunity")) AND (de:(calves OR "calf feeding") OR ti:(calf OR calves OR calf-cow OR "newborn beef" OR (neonat* NEAR/2 calves)) OR ab:(calf OR calves OR calf-cow OR "newborn beef" OR (neonat* NEAR/2 calves))) yr:[2003 TO 2023]) AND (((language:(("English"))) (NOT (item-type:(("Book chapter"))))))
