# Supplementary material for: Failure of Passive Immune Transfer in Neonatal Beef Calves: A Scoping Review
Source: Animals (Basel). 2025 Jul 14;15(14):2072. doi: 10.3390/ani15142072 (PMC12291800; doi:10.3390/ani15142072)
Supplement: Supplementary file 1 [file animals-15-02072-s001.zip › Table S2 Pubmed search.pdf]

Table S2: Search string for PubMed database for a scoping review on failure of passive immune transfer in neonatal beef calves. Search terms were developed with a combination of terms related to the study population, the interventions colostrum and vaccination, and the outcome passive immunity, as well as a filter for publication year.

Pubmed 227 results (search was repeated in January 2025 yielding additional results)

**Theme search**	**Synonyms (keywords)**
**#1 calves**	**“calf”[tiab] OR “calves”[tiab] OR “calf-cow”[tiab] OR “newborn beef”[tiab] OR “neonat* calves”[tiab:~2]**
**#2 colostrum**	**“colostrum”[tiab] OR “IgG”[tiab] OR “immunoglobulins G”[tiab] OR “immune globulins G”[tiab] OR “suckling”[tiab] OR “beef-suckler”[tiab] OR “Immunoglobulin G”[Mesh] OR "Colostrum"[Mesh]**
**#3 passive immunity**	“passive immun*”[tiab] OR “passive transfer”[tiab] OR "Immunity, Maternally-Acquired"[Mesh]
**#4 beef**	**“Beef” [tiab]OR “veal” [tiab]OR “Angus”[tiab] OR “Ayrshire”[tiab] OR “Boran”[tiab] OR “Brahman”[tiab] OR “Brangus”[tiab] OR “Braunvieh”[tiab] OR “Charolais”[tiab] OR “Fleckvieh”[tiab] OR “Friesian”[tiab] OR “Gelbvieh”[tiab] OR “Gir”[tiab] OR “Hereford”[tiab] OR “Holstein”[tiab] OR “Jersey”[tiab] OR “Limousin”[tiab] OR “Longhorn”[tiab] OR “Nellore”[tiab] OR “Ongole” OR “Sahiwal”[tiab] OR “Sanga”[tiab] OR “Shorthorn”[tiab] OR “Simmental”[tiab] OR “Wagyu”[tiab]**
**#5 vaccination**	**“neonatal vaccin*”[tiab] OR “beef calf vaccin*”[tiab] OR “prepartum vaccin*”[tiab]**
**#6**	**#5 OR #2**
**#7 227**	**#6 AND #4 AND #3 AND #1**
**#8 168**	**#7 AND english AND 2003-2023**
**Copy and paste**	**(("neonatal vaccin*"[Title/Abstract] OR "prepartum vaccin*"[Title/Abstract] OR ("Colostrum"[Title/Abstract] OR "IgG"[Title/Abstract] OR "immunoglobulins G"[Title/Abstract] OR "suckling"[Title/Abstract] OR "beef-suckler"[Title/Abstract] OR "Immunoglobulin G"[MeSH Terms] OR "Colostrum"[MeSH Terms])) AND ("Beef"[Title/Abstract] OR "veal"[Title/Abstract] OR "Angus"[Title/Abstract] OR "Ayrshire"[Title/Abstract] OR "Boran"[Title/Abstract] OR "Brahman"[Title/Abstract] OR "Brangus"[Title/Abstract] OR "Braunvieh"[Title/Abstract] OR "Charolais"[Title/Abstract] OR "Fleckvieh"[Title/Abstract] OR "Friesian"[Title/Abstract] OR "Gelbvieh"[Title/Abstract] OR**

"Gir"[Title/Abstract] OR "Hereford"[Title/Abstract] OR "Holstein"[Title/Abstract] OR "Jersey"[Title/Abstract] OR "Limousin"[Title/Abstract] OR "Longhorn"[Title/Abstract] OR "Nellore"[Title/Abstract] OR "Ongole"[All Fields] OR "Sahiwal"[Title/Abstract] OR "Sanga"[Title/Abstract] OR "Shorthorn"[Title/Abstract] OR "Simmental"[Title/Abstract] OR "Wagyu"[Title/Abstract]) AND ("passive immun*"[Title/Abstract] OR "passive transfer"[Title/Abstract] OR "immunity, maternally acquired"[MeSH Terms]) AND ("calf"[Title/Abstract] OR "calves"[Title/Abstract] OR "calf-cow"[Title/Abstract] OR "newborn beef"[Title/Abstract] OR "neonat calves"[Title/Abstract:~2])) AND ((2003/1/1:2023/12/31[pdat]) AND (english[Filter]))
