# Supplementary material for: Failure of Passive Immune Transfer in Neonatal Beef Calves: A Scoping Review
Source: Animals (Basel). 2025 Jul 14;15(14):2072. doi: 10.3390/ani15142072 (PMC12291800; doi:10.3390/ani15142072)
Supplement: Supplementary file 1 [file animals-15-02072-s001.zip › Table S3 Scopus search.pdf]

Table 3: Search string for Scopus database for a scoping review on failure of passive immune transfer in neonatal beef calves. Search terms were developed with a combination of terms related to the study population, the interventions colostrum and vaccination, and the outcome passive immunity, as well as a filter for publication year.

Scopus results 243 (search was repeated in January 2025 yielding additional results)

**#1 calves**	TITLE-ABS-KEY(**calf OR calves OR calf-cow OR "newborn beef" OR "neonat* calves" OR "neonat* calf")**
**#2 colostrum**	TITLE-ABS-KEY(**"colostrum" OR "IgG" OR "immunoglobulins G" OR "immune globulins G" OR suckling OR beef-suckler)**
**#3 passive immunity**	TITLE-ABS-KEY("passive immun*" OR "passive transfer")
**#4 beef**	TITLE-ABS-KEY(**Beef OR veal OR Angus OR Ayrshire OR Boran OR Brahman OR Brangus OR Braunvieh OR Charolais OR Fleckvieh OR Friesian OR Gelbvieh OR Gir OR Hereford OR Holstein OR Jersey OR Limousin OR Longhorn OR Nellore OR Ongole OR Sahiwal OR Sanga OR Shorthorn OR Simmental OR Wagyu)**
**#5 vaccination**	**(TITLE-ABS("neonatal vaccin*" OR "beef calf vaccin*" OR "prepartum vaccin*")**
**#6**	**#5 OR #2**
**#7 324**	**#6 AND #4 AND #3 AND #1**
**#8 243**	
**Copy and paste**	(TITLE-ABS-KEY (calf OR calves OR calf-cow OR "newborn beef" OR "neonat* calves" OR "neonat* calf")) AND (TITLE-ABS-KEY ("passive immun*" OR "passive transfer")) AND (TITLE-ABS-KEY (beef OR veal OR angus OR ayrshire OR boran OR brahman OR brangus OR braunvieh OR charolais OR fleckvieh OR friesian OR gelbvieh OR gir OR hereford OR holstein OR jersey OR limousin OR longhorn OR nellore OR ongole OR sahiwal OR sanga OR shorthorn OR simmental OR wagyu)) AND ((TITLE-ABS-KEY ("neonatal vaccin*" OR "beef calf vaccin*" OR "prepartum vaccin*") OR TITLE-ABS-KEY ("colostrum" OR "IgG" OR "immunoglobulins G" OR "immune globulins G" OR suckling OR beef-suckler))) AND (LIMIT-TO (PUBYEAR , 2023) OR LIMIT-TO (PUBYEAR , 2022) OR LIMIT-TO (PUBYEAR , 2021) OR LIMIT-TO (PUBYEAR , 2020) OR LIMIT-TO (PUBYEAR , 2019) OR LIMIT-TO (

PUBYEAR , 2018) OR LIMIT-TO (PUBYEAR , 2017) OR LIMIT-TO (PUBYEAR , 2016) OR LIMIT-TO (PUBYEAR , 2015) OR LIMIT-TO (PUBYEAR , 2014) OR LIMIT-TO (PUBYEAR , 2013) OR LIMIT-TO (PUBYEAR , 2012) OR LIMIT-TO (PUBYEAR , 2011) OR LIMIT-TO (PUBYEAR , 2010) OR LIMIT-TO (PUBYEAR , 2009) OR LIMIT-TO (PUBYEAR , 2008) OR LIMIT-TO (PUBYEAR , 2007) OR LIMIT-TO (PUBYEAR , 2006) OR LIMIT-TO (PUBYEAR , 2005) OR LIMIT-TO (PUBYEAR , 2004) OR LIMIT-TO (PUBYEAR , 2003)) AND (LIMIT-TO (LANGUAGE , "English"))
